# Energy consumption of ultrasound devices during routine applications and opportunities to save energy and costs

**DOI:** 10.1007/s00330-025-11822-8

**Published:** 2025-07-15

**Authors:** Rasid Mujkic, Karin Haller, Christian Kollmann

**Affiliations:** 1 Department of General Radiological Diagnostics, University Hospital Graz, Radiology, Auenbruggerplatz 9, 8036 Graz, Austria; 2 University of Applied Sciences, Department of Radiological Technology, Johannes Gutenberg-Straße 3, 2700 Wiener Neustadt, Austria; 3 Center for Medical Physics & Biomedical Engineering, Medical University Vienna, Waehringer Guertel 18-20, 190 Vienna, Austria

**Keywords:** Ultrasonography, Cost saving, Sustainability, Diagnostic equipment, Medicine

## Abstract

**Objective:**

To determine the real energy consumption, including the idle and off-mode status of ultrasound devices and their multiparametric modes during daily examinations.

**Material and methods:**

In this study, the electrical power consumption from 9 ultrasound devices and 18 probes used in routine medical practice was measured for a full 24 h over a week. The equipment was located in the neurology and cardiology departments of a large hospital, as well as in a radiology medical practice. The devices were manufactured by different companies and varied in age.

**Results:**

Short-term measurements recorded the electrical power consumption during patient scans, revealing real power values for the applied modalities, which ranged between a mean of 182.5 W and 415.5 W, depending on the device and application field. Long-term measurements, reflecting the typical working days of these departments, showed total annual electrical power consumption ranging from 344.6 kWh to 1122.6 kWh. Energy-saving possibilities are discussed, highlighting that reducing idle-mode durations can significantly lower electrical costs by saving energy of 3–16%.

**Conclusion:**

This study is the first to document in detail the power consumption of different ultrasound devices and modalities, including idle and off-mode statuses, during routine use.

**Key Points:**

***Question*** *Which procedures during ultrasound examinations have high power consumption and what is the idle- and off-mode consumption of different devices.*

***Findings*** *Energy consumption can vary greatly depending on the application. Savings potentials of 3-16% could easily be realized in the daily routine.*

***Clinical relevance*** *Exact knowledge of power consumption of a device during off-, idle-, and some often-used active-modes (all are easy and fast to perform) can help to optimize examination scenarios and minimize power consumption and thus energy costs.*

**Graphical Abstract:**

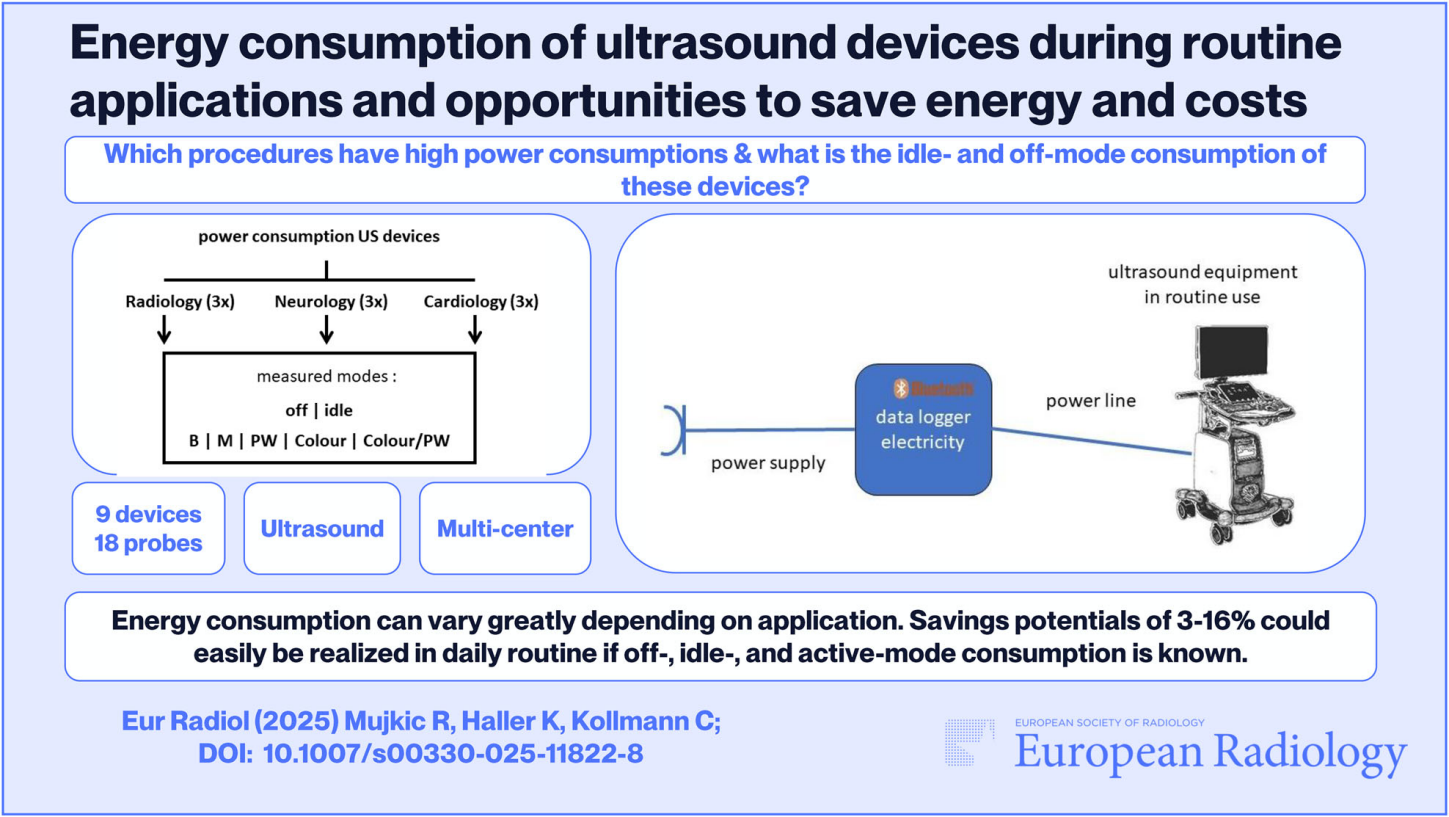

## Introduction

Hospitals are huge energy consumers, operating 24/7 throughout the year to maintain energy-intensive infrastructure, including heating, cooling and ventilation systems, computing, archiving, sterilization, refrigeration, laundry, food services, and the use of medical and laboratory equipment [1, 2]. Electrical energy consumption alone accounts for 30–50% of total building energy use [3, 4], with medical imaging equipment contributing approximately 5% [5].

Within hospitals, the radiology department plays a substantial role in electrical energy consumption due to its use of MRI, CT, and X-ray machines.

An MRI scanner typically consumes more than twice the energy of a CT scanner and ten times the energy of an X-ray scanner during operation [4, 5]. Rising electricity costs and growing awareness of the environmental impact of healthcare services have brought energy-saving measures and sustainability into focus [2, 4, 6–11].

In 2008, the European Commission established a regulation for energy savings and ecodesign requirements for electrical and electronic household and office equipment (European Commission regulation #1275/2008). This regulation aimed to significantly reduce electricity consumption in standby and off-mode functionalities for new products. In 2005, these modes alone accounted for 47 TWh of energy consumption in the European Community, with projections of further increases in subsequent years [12].

For MRI and CT equipment, several studies have analyzed real-time energy consumption at various stages of operation [4, 6, 13–15]. However, for ultrasound devices, energy consumption data are often limited to manufacturer estimates or unspecified in real equipment use [9, 14, 16].

This study analyzes the real-time electrical energy consumption of ultrasound devices and probes routinely used in a hospital’s cardiology and neurology departments, as well as in radiology practices. Both long-term and short-term measurements were conducted, providing detailed data for each ultrasound mode used during patient scans. Based on real working-day data, annual energy consumption was estimated, and potential energy-saving measures were identified.

## Materials and methods

In this study, 9 ultrasound devices and 18 probes (Table 1) from different manufacturers and varying ages have been included. The equipment was installed in the neurology and cardiology departments of a large hospital (LKH Graz, Austria, approximately 1500 beds) and in a radiology medical practice in Graz, Austria. While the hospital departments primarily focus on examinations using Doppler modes, the medical practice takes an interdisciplinary approach, employing a mix of B- and Doppler modes.

**Table 1:** Summary of involved ultrasound devices and probes

	Device type	YOM	SWV	Probes	Application
Radiology1	GE LogiQ S8 R4 XD Clear 2.0	12/2020	R4 2.3	C1-6-D; 9L-D; ML6-15-D	Interdisciplinary
Radiology2	GE LogiQ S8 R4 XD Clear 2.0	02/2018	R4 2.3	C1-6-D; 9L-D; ML6-15-D	Interdisciplinary
Radiology3	GE LogiQ S8 R4 XD Clear 2.0	10/2017	R4 2.3	C1-6-D; 9L-D; ML6-15-D	Interdisciplinary
Neurology1	GE Vivid E90	08/2022	204.97.0	M5Sc-D; 9L-D	Cardiology
Neurology2	GE Vivid E90	10/2017	204.85.0	M5Sc-D; 9L-D	Cardiology
Neurology3	GE LogiQ Fortis	06/2022	R3 1.1	M5Sc-D; L2-9-D	Interdisciplinary
Cardiology1	Philips EpiQ CVx	04/2020	R 7.0.5	X5-1	Cardiology
Cardiology2	Siemens Acuson SC2000	01/2018	VB 21D	4V1-C	Cardiology
Cardiology3	GE Vivid E95	07/2022	204.91.0	4Vc-D	Cardiology

*YOM* year of manufacture, *SWV* software version

Consent for this study and for the collection of electrical data was obtained from all department heads and the medical practice owner.

**Fig. 1:**
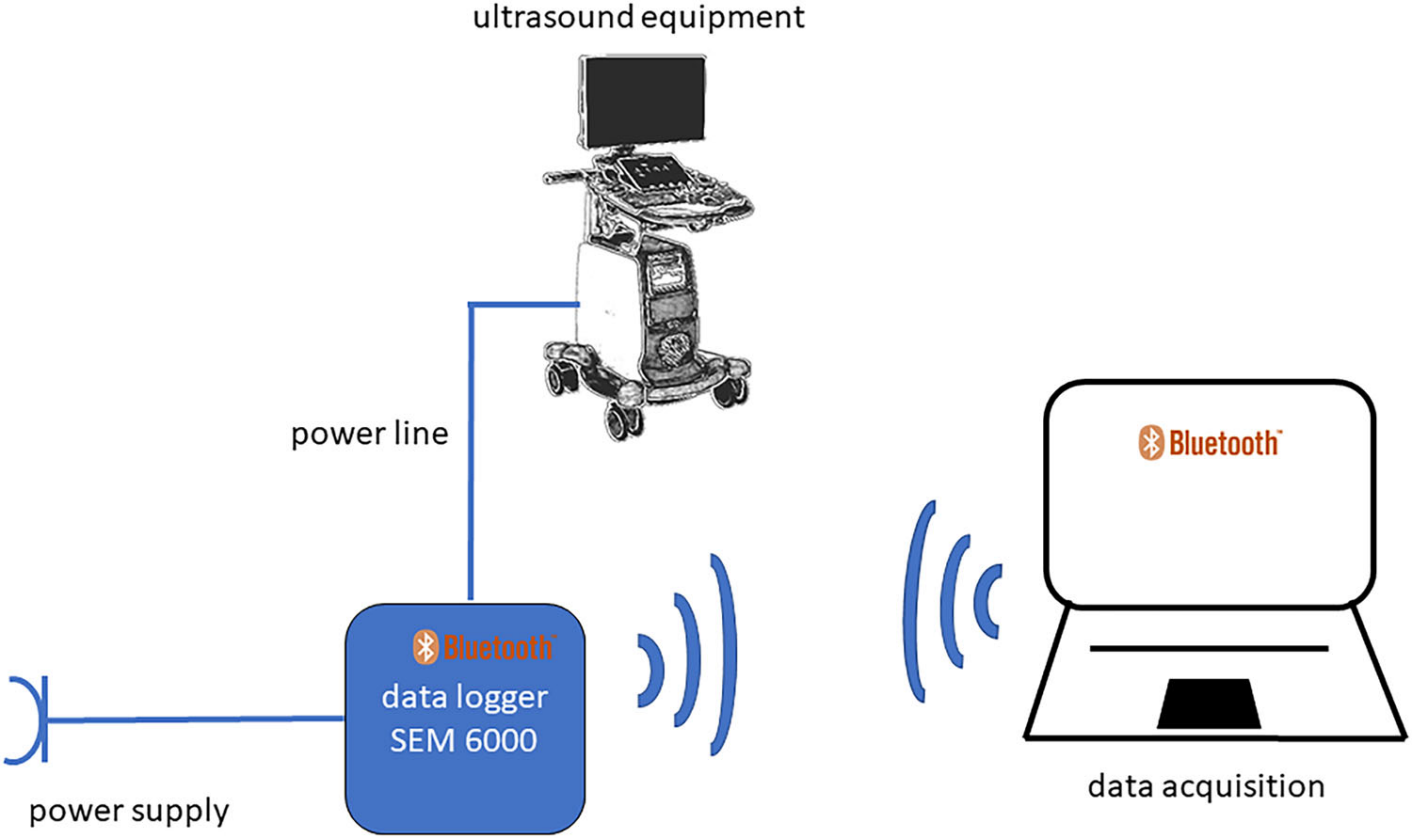
Experimental setup for power measurements

The electrical power data were measured using an electrical data logger (Voltcraft SEM6000, Conrad Electronic, part #1558906-62) paired with modified software to transmit data via Bluetooth 4.0 to a notebook (MacBook Air vers. 2020) running a virtual machine under VMware Fusion (vers.13.5.0, Broadcom) with Linux OS (Ubuntu vers. 22.04.3, 64-bit). The data logger and software enabled the recording of the following electrical parameters with high temporal resolution (every 1–2 s): voltage (V), actual/maximal current (A), power (W), power frequency (Hz) and power factor. Measurements were performed with an accuracy range of ± 1–10%. All equipment was connected sequentially to the data logger, which maintained the connection to the power supply and facilitated data exchange with the notebook (Fig. 1).

### Energy measurement procedures

For each probe, the actual energy consumption was measured once it was activated for a specific modality (e.g., B-mode, Doppler, continuous wave-mode) along with the device’s energy consumption in idle-mode (e.g., standby or inactive) and off-mode (connected to the power line only) for each of the 9 devices (Table 2).

**Table 2:** Power (W) consumption averaged over all probes for separate modes and off- and idle-mode per ultrasound device

		Radiology			Neurology			Cardiology		
		R1	R2	R3	N1	N2	N3	C1	C2	C3
Off-mode		4	4	4	16	16.5	0.45	57	52	16
Idle-mode		163.5	170.8	176.1	368.9	377.9	232.9	339.9	580.9	347.6
Savings (%) off- vs. idle-mode		97.6	97.7	97.7	95.7	95.6	99.8	83.2	91.1	95.4
Active-mode	B	193.7	192.2	186.2	388.3	414.6	252.9	380.3	644.6	384.2
	M	187.0	194.8	182.5	392.5	415.5	253.6	385.2	645	381.4
	PW	191.6	192.5	191.9	380.1	398.2	256.1	358.4	645	379.1
	Color	191	191.5	187.6	381.6	412.4	256.8	383.5	668.1	401.2
	Color/PW	193.3	188.8	193.1	385.6	399.5	259.5	383.2	638.6	379.3
Savings (%) idle- vs. active-mode		12.6–15.6	9.5–12.3	3.5–8.8	2.9–6	5.1–9	7.9–10.3	5.2–11.8	9–13.1	8.3–13.4

*B* B-Mode, *M* M-Mode, *PW* pulsed-wave-mode,* Color* color-mode, *Color/PW* combined color and PW-mode

Additionally, the energy values during routine use in the three different medical application fields were documented as short-term measurements (STM) over 1 h for selected examinations conducted during a working week. The distribution of modalities used during these applications was also recorded. Long-term measurements (LTM) were logged over 24 h for a duration of 7 days for each device. While STM data were acquired at nearly 1-s intervals, LTM data were aggregated over a working day by the data logger and downloaded as Excel-compatible files after one week.

The STM data were primarily used to calculate the average power consumption for each modality (Table 2). In contrast, the LTM data provided a more reliable basis for estimating the annual energy consumption of each ultrasound device (Table 3), as they captured the realistic working-day schedule (8 h active, 16 h off-time) and weekend procedures (offline for radiology/neurology; disconnected from the power line for cardiology) in the ambulances.

**Table 3:** Daily and annual energy consumption based on LTM data

	Avg. energy consumption (kWh)			
	Per day	Annual (extrapolated, mean)		
	LTM	LTM (253 days)	LTM (365 days)	Diff (abs (kWh)/%)
Radiology1	1320	333,875	344,627	10.8/3.2
Radiology2	2497	631,741	642,493	10.8/1.7
Radiology3	2755	696,964	707,716	10.8/1.5
Neurology1	2839	718,267	761,275	43/6
Neurology2	1672	422,915	467,267	44.4/10.5
Neurology3	-	-	-	-
Cardiology1	3172	802,415	802,415	0/0
Cardiology2	4437	1,122,612	1,122,612	0/0
Cardiology3	2275	575,677	575,677	0/0

*LTM* long-term measurement

The daily averages were extrapolated to reflect 253 working days per year, providing accurate energy consumption data for the specific workflows in Austria. To enable comparison with other literature, the data were also expanded to a full calendar year (365 days) using the same working schedule.

## Results

The averaged power data from STM measurements for each ultrasound method are presented in Table 2. Identical devices in the radiology department show similar consumption data, regardless of their differing manufacturing ages, as they use the same software version (Table 1). In the neurology department, two devices (N1, N2) have identical construction but different software versions. The device with the newer software version (N1) demonstrates slightly lower power consumption in all modes (range 3.5–7.5%) compared to the other device (N2), while a third system, which has a different construction, consumes less power in all modes.

In the cardiology department, all three devices are of different designs but exhibit the highest power consumption in both off-mode and active-mode values in the table. Across all machines, power consumption in idle-mode (when the ‘freeze’ button is off) is approximately 3-16% lower than in active-mode.

**Fig. 2:**
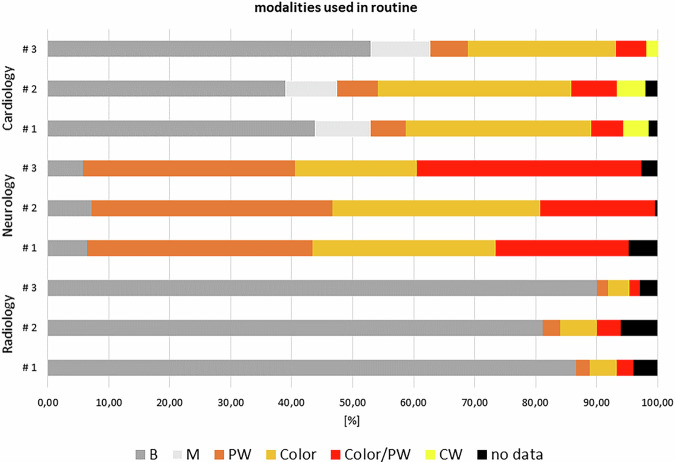
Proportion of modalities used during examinations (averaged, in percentage)

**Fig. 3:**
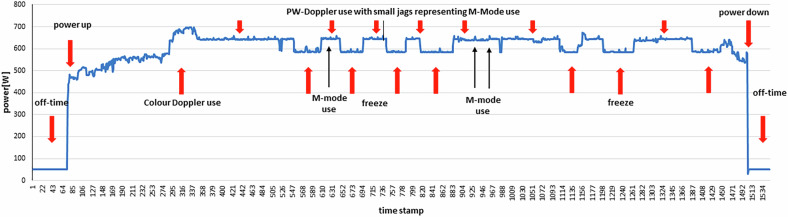
Energy consumption during an ultrasound examination (example from Cardiology 2)

The off-mode data show the lowest values measured and, compared with the idle-mode values, this is a tremendous reduction in electrical energy consumption of 83.2–99.8% depending on the device (Table 2). B-mode examinations dominated in the radiology field, while Doppler modes were primarily applied in cardiology and neurology (Fig. 2). Interestingly, the neurology department, specialized in stroke diagnostics and intra- and extra-cranial vessel examinations, had the highest rate of Doppler mode usage—not the cardiology department. The data were extracted from STM-logged power values, which also included unassignable segments corresponding to switching times between modalities (black segments in Fig. 2). A complete STM data set of power consumption is shown in Fig. 3, starting with booting, scanning a patient in cardiology (C2), and powering down the equipment. The low off-mode power values are clearly visible at the beginning and end of the timeline, followed by significant power consumption during booting and scanning. The active scanning phase includes different modes, mainly B-mode, Color Doppler, and occasionally PW-mode applications. Lower power dips during scanning represent standby times (freeze mode), manually activated by the examiner (marked in Fig. 3).

**Fig. 4:**
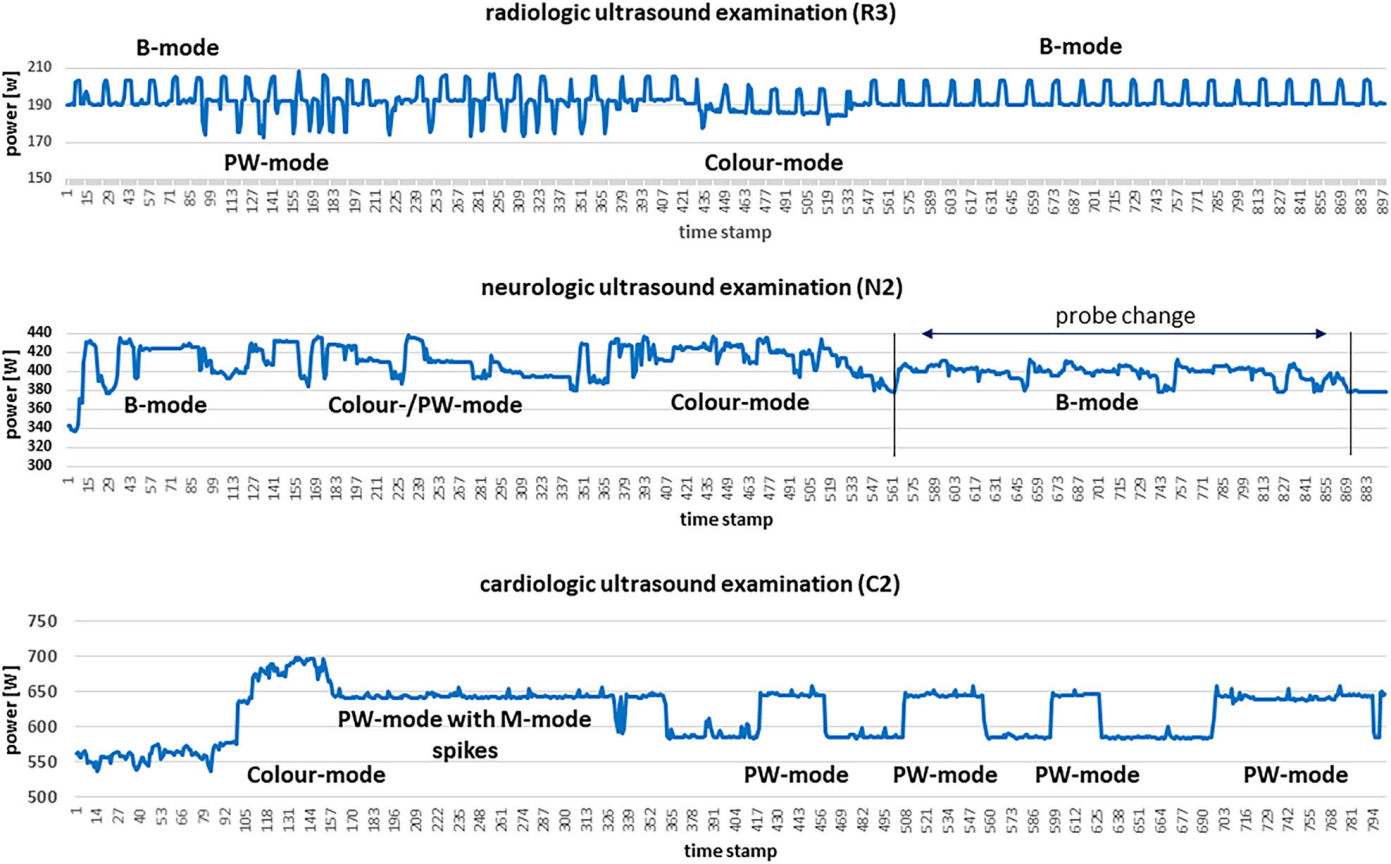
Distribution of power consumption for 3 different applications and identified scanning modes within an examination

The power distribution patterns during scans differ significantly across radiology, neurology, and cardiology (Fig. 4). Each panel represents a full-length routine patient scan, highlighting prominent scanning mode phases.

- In radiology, the examination shows numerous B-mode spikes, reflecting the rapid probe movement over the abdomen, followed by dips associated with PW-mode activation (Fig. 4, top).
- In neurology, power consumption is more complex to interpret due to frequent mode changes within short intervals, as well as a probe change during scanning. Generally, these scans operate at a higher power level (Fig. 4, middle).
- In cardiology, the scans involve longer periods of a single mode. The highest power consumption occurs during Color Doppler use, with lower values observed during standby/freeze intervals between PW-mode sessions. A slight increase in power consumption during M-mode (spikes) is noticeable within PW-mode intervals (Fig. 4, bottom).

The LTM data, representing average energy consumption over one working day, and the extrapolated values for a working year and calendar year, are summarized in Table 3.

For cardiologic applications, the equipment demonstrates high energy consumption, though some devices from other departments also show significant energy use (comparison: domestic freezer: 415 kWh/yr; refrigerator: 330 kWh/yr, and electrical cooker: 445 kWh/yr [17]). The ultrasound system in the neurology department (Neurology3) is not included in the analysis, as it is only sporadically used for interdisciplinary bedside examinations. Consequently, STM data were recorded, but no LTM data were acquired.

The last column of Table 3 presents the differences between the two annual LTM datasets in absolute and percentage values. These differences reflect energy consumption over the year if devices remain connected to the power line in off-mode during weekends and national holidays. In contrast, cardiology departments disconnect their machines these days, ensuring that their total energy consumption does not increase during off-times.

**Fig. 5:**
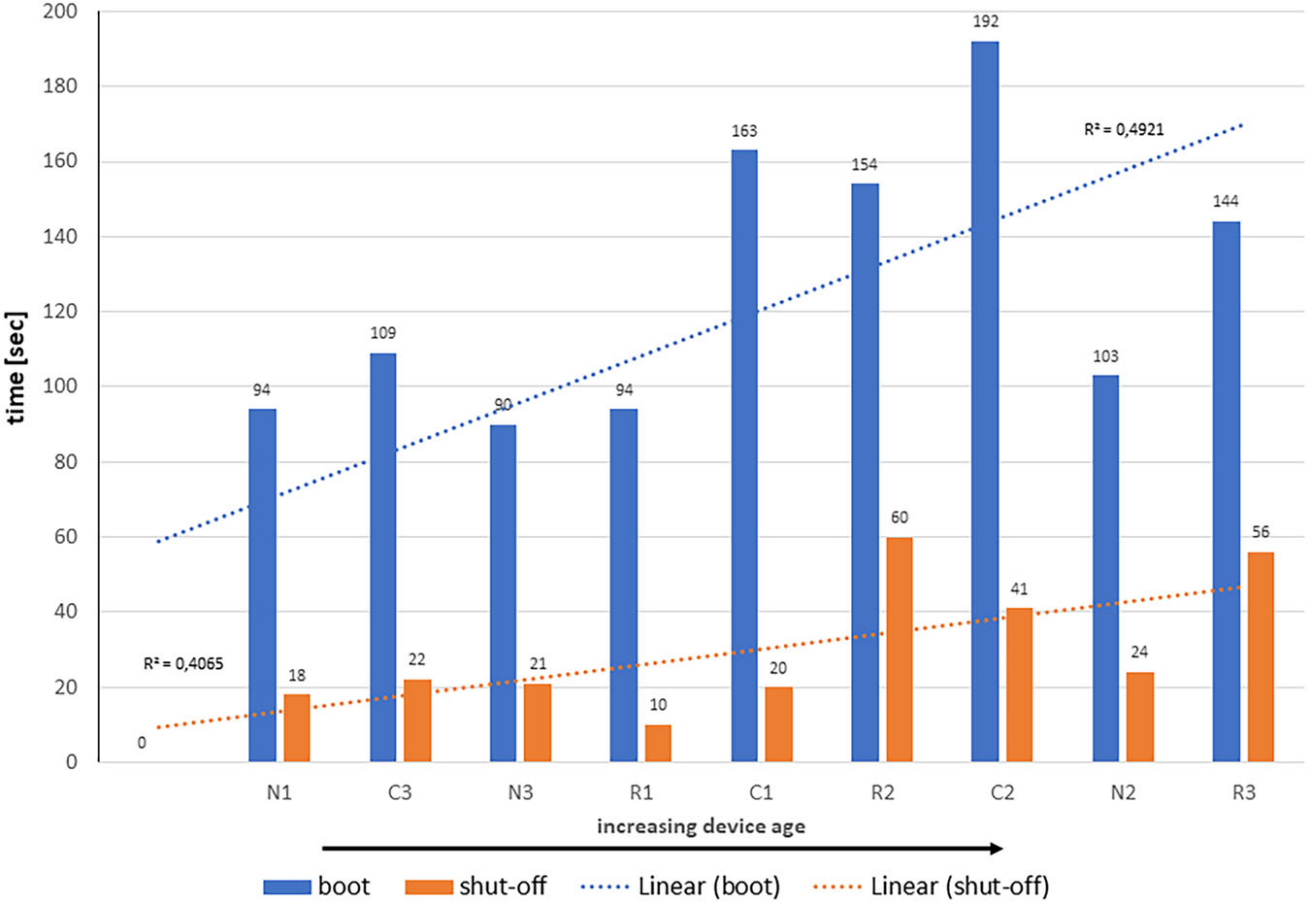
Duration for booting and shut-off phase (in seconds) of the involved ultrasound devices

Another important aspect of routine use is the booting and shutdown duration of ultrasound systems. Figure 5 illustrates these times, measured in relation to the equipment’s manufacturing dates. Generally, the booting time is longer than the shutdown time. A weak correlation between equipment age and duration can be observed for both booting and shutdown times. However, the exact durations vary depending on the individual configuration of each ultrasound device, which includes integrated hardware and software components.

## Discussion

This study analyzed the energy consumption of ultrasound devices used for specialized neurological, cardiological, radiological, and interdisciplinary applications. The collected data reflect the individual patterns of examiners, their real-world usage, and the technical specifications of the devices within these departments. To our knowledge, this is the first study to present real-world energy consumption data from routine patient scans in the investigated medical fields with these details. This is important for department chiefs or health-care managers to optimize the workflow and utilization of devices or to make improvements in energy with software upgrades or by introducing new equipment generation.

Significant energy savings can be achieved by powering down ultrasound devices after a working day instead of leaving them in idle-mode (standby), where the device remains inactive but continues to consume energy. In practice, users sometimes forget to press the freeze button at the end of a patient scan or fail to comply with energy-saving protocols [18]. As a result, the equipment continues to operate unnecessarily, consuming large amounts of energy that is primarily released as heat, increasing the room temperature. This issue can be addressed by configuring the device to switch automatically to idle-mode after several minutes of inactivity. This adjustment alone can reduce energy consumption by approximately 83–98%, depending on the machine (Table 2). Similar differences in energy consumption have been observed for other medical systems, such as MRI, PET/CT, CT, dual-energy CT, and anesthesia machines [6–8, 13, 19, 20].

Additional but smaller energy savings of 1.5–10.5% (Table 3) can be realized by disconnecting devices from the power line after a working day. To enhance sustainability, only machines required for emergency or unplanned scans should remain in standby mode. Furthermore, the adoption of newer equipment generations or updated software versions can contribute to reduced energy consumption. Modern hardware and software are designed to optimize energy efficiency during imaging and scanning processes. Our findings highlight factors affecting energy reduction in newer device generations and differences between manufacturers and application fields. For example, the boot and shutdown times of newer ultrasound machines with identical construction (e.g., R1, R3; N1, N2 in Fig. 4) are shorter, potentially due to fewer energy-intensive hardware modules (e.g., R1, R3) or optimized software control (e.g., N1, N2). In general, booting duration depends on the number and configuration of software modules installed and the time required for internal electronic maintenance checks before the system becomes ready to scan.

The STM data (Table 2) were derived from real patient examinations across various medical fields, incorporating standardized scanning procedures. However, these procedures naturally vary depending on the user, their individual settings, and the chosen presets of the equipment. As seen in Fig. 3 and Fig. 4, the temporal electrical current fluctuates continuously due to real-time examination needs, influenced by user handling, settings, and the duration of the selected scanning modes.

During routine work, it was challenging to collect specific STM data from patient scans. LTM data were recorded over a defined period (1 week), which does not account for specific seasonal fluctuations in examination volume throughout the year but might be representative of normal workload. Similarly, off-mode consumption was considered within a fixed framework, without factoring in potential variations due to differing medical working days or maintenance conditions. Therefore, while the generalizability of the results to other medical departments or ultrasound devices from different manufacturers is limited, this study provides, to our knowledge, the first detailed insight into energy consumption during routine medical use.

To compare the total annual energy consumption of an ultrasound device with other medical imaging equipment, LTM data were extrapolated to a full calendar year (365 days, Table 3) and presented alongside energy-related information from the literature (Table 4).

**Table 4:** Average energy consumption of medical equipment based on literature

	Ultrasound	CT^a^	MRI^a^	PET/CT^b^	SPECT^c^	X-ray^d^
avg. consumption/exam (kWh)	0.043–0.175	0.7–1.9	15–25	3.23	8.21^e^	-
avg. exam time (min)	8–25	16	45–51	20	60	-
avg. consumption/day (kWh)	1.3–4.4	50–92	363–530	77.5^e^	65.7	87.34–130.83^e^
avg. consumption/anno (kWh)	344–1123	78,679	134,037–149,655	76,741	16,622^e^	22,097–33,100^e^

*avg* average, *CT* Computer tomography, *MRI* magnetic resonance imaging

^a^ From Heye et al

^b^ From Artz

^c^ From Marwick et al

^d^ From Esmaeili et al

^e^ Derived from source data

Ultrasound imaging machines consume only a small fraction of yearly electrical energy compared to other clinical imaging systems [4, 6, 7, 16, 21]. However, modern hospitals typically have numerous ultrasound devices across various departments and ambulances, most of which remain constantly connected to the power line. Consequently, the total energy consumption of this imaging modality should not be underestimated. With this yearly energy consumption, you could drive an electric Tesla Model 3 car (2024) between 24.6–80.2 km (World harmonized light vehicles test procedure cycle: 14 kWh/100 km [22]).

Healthcare managers increasingly prioritize energy efficiency and long-term electrical costs when replacing equipment or making new procurements [10]. Efficient energy management is particularly critical for mobile or wearable medical devices [23–26] and advancements in this area will influence future generations of ultrasound machines, making them more energy-efficient.

## Abbreviations

B: Brightness-mode
CT: Computer-tomography
LTM: Long-term measurements
M: Motion-mode
MRI: Magnetic resonance imaging
PET: Positron emission tomography
PW: Pulsed-wave mode
STM: Short-term measurements
